# Anti-Epileptic Effect of Crocin on Experimental Temporal Lobe Epilepsy in Mice

**DOI:** 10.3389/fphar.2022.757729

**Published:** 2022-03-31

**Authors:** Kai Zhong, Chengyu Qian, Rui Lyu, Xinyi Wang, Zhe Hu, Jie Yu, Jing Ma, Yilu Ye

**Affiliations:** ^1^ Department of Pharmacology, School of Basic Medical Sciences and Forensic Medicine, Hangzhou Medical College, Hangzhou, China; ^2^ College of Basic Medical Science, Zhejiang Chinese Medical University, Hangzhou, China; ^3^ Department of Pharmacy, Xinhua Hospital, Shanghai Jiaotong University School of Medicine, Shanghai, China

**Keywords:** temporal lobe epilepsy, crocin, kindling, pilocarpine, spontaneous recurrent seizure

## Abstract

Temporal lobe epilepsy (TLE) is a common kind of refractory epilepsy. More than 30% TLE patients were multi-drug resistant. Some patients may even develop into status epilepticus (SE) because of failing to control seizures. Thus, one of the avid goals for anti-epileptic drug development is to discover novel potential compounds to treat TLE or even SE. Crocin, an effective component of *Crocus sativus L.*, has been applied in several epileptogenic models to test its anti-epileptic effect. However, it is still controversial and its effect on TLE remains unclear. Therefore, we investigated the effects of crocin on epileptogenesis, generalized seizures (GS) in hippocampal rapid electrical kindling model as well as SE and spotaneous recurrent seizure (SRS) in pilocarpine-induced TLE model in ICR mice in this study. The results showed that seizure stages and cumulative afterdischarge duration were significantly depressed by crocin (20 and 50 mg/kg) during hippocampal rapid kindling acquisition. And crocin (100 mg/kg) significantly reduced the incidence of GS and average seizure stages in fully kindled animals. In pilocarpine-induced TLE model, the latency of SE was significantly prolonged and the mortality of SE was significantly decreased by crocin (100 mg/kg), which can also significantly suppress the number of SRS. The underlying mechanism of crocin may be involved in the protection of neurons, the decrease of tumor necrosis factor-α in the hippocampus and the increase of brain derived neurotrophic factor in the cortex. In conclusion, crocin may be a potential and promising anti-epileptic compound for treatment of TLE.

## Introduction

Although several new anti-epileptic drugs (AEDs) have been developed, still 30% patients (even 75% in mesial TLE) remain resistant to many kinds of AEDs and gradually progress to refractory epilepsy (Wang et al., 2018; Wang and Chen, 2019; Xu et al., 2021a). Some patients may even develop into status epilepticus (SE) because of failing to control seizures. For these patients, epileptic foci resection surgery is an available treatment; however, it is not suitable for many cases due to unacceptable neurological or cognitive impairments (Wang et al., 2021). Therefore, it is essential for seeking novel effective AEDs.

Natural products from traditional Chinese medicine have emerged to be a promising choice for the treatment of epilepsy. Several natural products have been reported to be effective on experimental epilepsy and associated neurobehavioral comorbidities (Sucher and Carles, 2015; Bo-Qiang et al., 2018). Saffron, the dried stigmas of *Crocus sativus L. (*Iridaceae*)*, is being widely used in traditional medicine for a wide variety of neurological conditions (Singh, 2015). Crocin is considered as one of the main effective components of saffron with a low toxicity (Bostan et al., 2017). Extensive preclinical and clinical studies about its traditional use have been reported, such as anti-inflammation (Teng et al., 2021), anti-tumor (Mousavi et al., 2009), sedation and hypnosis (Masaki et al., 2012), anti-depression (Amin et al., 2015), anti-anxiety (Pitsikas et al., 2008) and anti-Parkinson’s disease (Rao et al., 2016). Several papers also reported its anti-epileptic activity in experimental epilepsy. Tamaddonfard et al. (2012) found crocin significantly inhibits epileptiform activities induced by penicillin. Recently, Mazumder et al. (2017) also reported that crocin can attenuate pentylenetetrazole (PTZ) kindling development. However, Hosseinzadeh and Talebzadeh (2005) found crocin is ineffective on PTZ induced seizures. Based on this controversy, it is necessary to further evaluate the anti-epileptic effect of crocin in more epileptic models (Alavizadeh and Hosseinzadeh, 2014).

In previous report, we have found that low-dose crocin can retard the progression in hippocampus rapid kindling acquisition in C57BL/6J mice, while high-dose crocin relieved the generalized seizures (GS) in fully-kindled mice (Wang et al., 2017a). The results suggested that the crocin may have a potential anti-epileptic effect in experimental TLE. However, two questions are still unclear. The first one is that the most patients suffer the spontaneous recurrent seizures (SRS), while the effect of crocin on SRS is still unknown. A pilocarpine model of mice, which is considered to mimic SE and SRS of patients, is always utilized to study the epileptogenesis and screen new AEDs (Zhong et al., 2012; Xu et al., 2016). So we first aim to investigate the anti-epileptic effect of crocin on the SE and SRS in pilocarpine-induced model in the present study. The second one is that although several hypotheses on the underlying anti-epileptic target of the crocin have been provided by some reports, it remains not very clear (Ahmed et al., 2020). Based on our preliminary findings, we speculate that brain derived neurotrophic factor (BDNF), or some inflammatory factors, such as interleukin-1β (IL-1β) and tumor necrosis factor-α (TNF-α), may be potential targets for the anti-epileptic effect of crocin. Hence, we also aim to explore the underlying anti-epileptic target of crocin in this study.

## Materials and Methods

### Animals

Male ICR mice were purchased from Shanghai SLAC Laboratory Animal Co., Ltd., weighing 25–35 g, raised at the Laboratory Animal Center of Hangzhou Medicine College. Mice were housed in individual cages with a 12-h light/dark cycle (lights on from 08:00 to 20:00). Water and food were provided ad libitum. Experiments were carried out between 10:00 and 17:00 in each day. All experiments were approved by the Hangzhou Medicine College Animal Experimentation Committee and were in complete compliance with the National Institutes of Health Guide for the Care and Use of Laboratory Animals.

### Surgery

Under 1% pentobarbital sodium anesthesia (40 mg/kg, i.p.), mice were mounted in a stereotaxic apparatus (512600, Stoelting, United States). Electrodes were implanted into the right hippocampus (AP: −2.9 mm, L: −3.0 mm, V: −3.0 mm) (Zhao et al., 2020). The electrodes were made of twisted stainless steel Teflon-coated wires (diameter 0.2 mm, A.M. Systems, United States) insulated except at the tip (0.5 mm); the tip separation was about 0.5 mm. The electrodes were connected to a miniature receptacle, which was attached to the skull with dental cement. After surgery, mice were allowed 7 days of recovery.

### Hippocampal Rapid Electrical Kindling Procedure

As described by the previous papers (Jin et al., 2013; Wang et al., 2017b), electrical stimulations were delivered by a constant-current stimulator (SEN-7203, SS-202J, Nihon Kohden, Japan) and electroencephalograms (EEG) were recorded with a Neuroscan system (Compumedics, Melbourne, Australia). The afterdischarge threshold (ADT) was determined by an application of a 2 s train of 1 ms monophasic square-waves at 20 Hz, beginning at 40 μA and increasing by 20% which were given at 1 min intervals until an electrographic afterdischarge (AD) lasting at least 5 s was elicited. Only the animals with an ADT less than 400 μA can be used for following experiments. Six hippocampal kindling stimulations (1 ms pulses, 20 Hz frequency, 2 s duration) at 30 min intervals with an intensity of 400 μA daily were delivered for 8 days.

Behavioral seizures were scored according to Racine’s scale (Racine, 1972), as modified for the mouse: stage 0, no response or behavior arrest; stage 1, chewing or facial twitches; stage 2, chewing and head nodding; stage 3, unilateral forelimb clonus; stage 4, bilateral forelimb clonus and rearing; stage 5, rearing and falling. Seizure stages 1–3 indicate focal seizures, while stages 4–5 are considered as GS (Engel et al., 1978). When mice exhibited three consecutive stage 5 seizures, they were regarded as fully kindled. In addition, the AD duration (ADD) was recorded as well.

### Assessment of the Effects of Crocin on Kindling Acquisition

**FIGURE 1:**
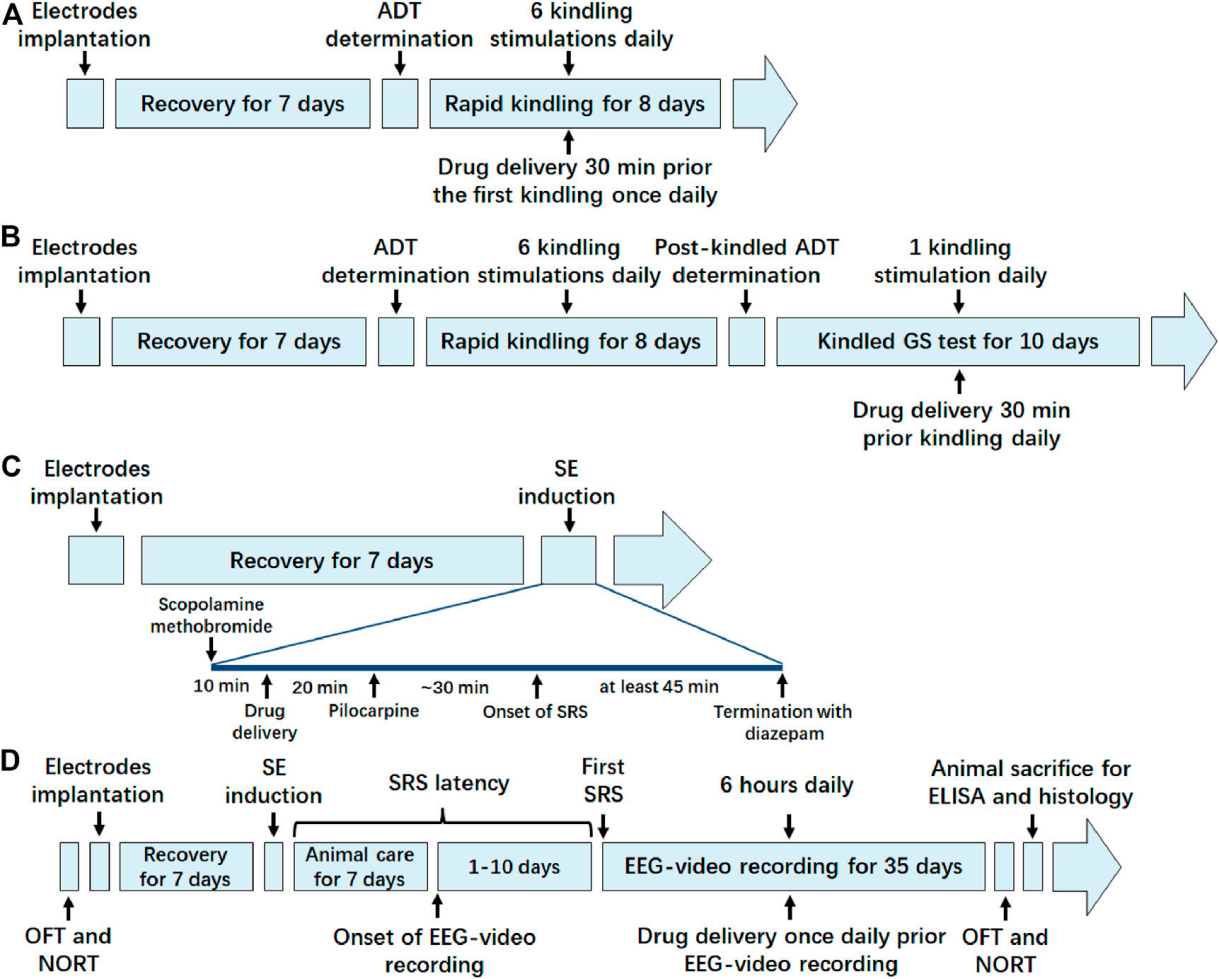
Schematic timeline diagram of experiments in each animal model. **(A)** Timeline of the experiment in hippocampal rapid kindling model; **(B)** timeline of the experiment in fully kindled model; **(C)** timeline of the experiment in pilocarpine-induced SE model; **(D)** timeline of the experiment in pilocarpine-induced SRS model. ADT, after discharge threshold; GS, generalized seizure; SE, status epilepticus; SRS, spontaneous recurrent seizure; OFT, open field test; NORT, novel object recognization test; EEG, electroencephalograms; ELISA, enzyme linked immunosorbent assay.

Mice were randomly divided into four groups matched for their ADTs. The initial ADTs of most mice ranged from 100 to 200 μA, thus there was no significant variability in the initial ADTs of mice in each group. In group 1–3, 10, 20 or 50 mg/kg crocin (Sigma-Aldrich, St. Louis, MO, United States) was respectively delivered i.p. 30 min prior the first electrical stimulation (methods and parameters were described above) of each day. In group 4, equivalent 0.9% saline (NS) was instead delivered as a vehicle control. Schematic timeline of the experiment is shown as Figure 1A. Seizure stages were recorded by two experimenters who were blinded to the grouping and drug delivery after each stimulation. ADD was subsequently calculated according to EEG recorded as described above. The number of stimulations required to reach and remain at each seizure stage was also calculated to analyze the stepwise progression of kindling seizures (Jin et al., 2013; Chen et al., 2020).

### Assessment of the Effects of Crocin on Fully Kindled GS

The post-kindled ADT of fully kindled mice (without any drug during kindling) was determined by the same procedure used for kindling ADT. On the next day, mice were divided into four groups. In group 1–3, 20, 50 or 100 mg/kg crocin (i.p.) was delivered 30 min prior the once-daily electrical stimulation (parameters were described above) for 10 days. In group 4, equivalent NS was instead delivered as a vehicle control. Schematic timeline of the experiment is shown as Figure 1B. The seizure stage and ADD were measured by the same procedures used for kindling seizures. GS duration (GSD) was also calculated from the onset to the termination of GS. In addition, the incidence of GS and average seizure stage during 10 days were calculated (Wang et al., 2017a).

### Assessment of the Effects of Crocin on Pilocarpine-Induced SE

Schematic timeline of the experiment is shown as Figure 1C. Mice with electrodes implantation at the hippocampus were randomly divided into four groups. Scopolamine methobromide (1 mg/kg, i.p., Sigma-Aldrich, St. Louis, MO, United States) was administrated 30 min before SE induction and then crocin (50, 100 or 200 mg/kg, i.p) or equivalent NS was given 20 min prior to pilocarpine. Pilocarpine (350 mg/kg, i.p., Sigma-Aldrich, St. Louis, MO, United States) was administrated to induce SE. When mice exhibited continuous tonic-clonic seizures and epileptic spikes in EEG, usually following with stage 4–5 seizures, they were defined as the onset of SE. The surviving mice with SE lasting at least 45 min were followed by diazepam (5 mg/kg, i.p. KingYork, TianJin, China) to terminate the SE (Curia et al., 2008; Xu et al., 2020). The mice, which did not experience stage 4 or 5 seizures following the pilocarpine injection or did not maintain the seizure intensity (less than 3 times stage 4 or 5 seizures in 45 min) were excluded. The latency of SE and the mortality of mice in each group were recorded by two experimenters who were blinded to the grouping and drug delivery.

### Assessment of the Effects of Crocin on Pilocarpine-Induced SRS

Schematic timeline of the experiment is shown as Figure 1D. SE was induced by pilocarpine as described above. After termination with diazepam, surviving mice were rehydrated with NS (4 ml/kg, i.p.) and given special care for the next 7 days by feeding powdered feed and water (i.g. if necessary). The untreated group was given an equal volume of NS instead of pilocarpine, and the rest of the operation was performed as above. From day 8 after induction of SE, SRS of the surviving mice were recorded by EEG-video for 6 h daily (11:00–17:00). SRS was defined as repetitive epileptic spikes with a frequency >5 Hz and a duration >20 s on EEGs recorded by a Powerlab system (AD Instruments, Bella Vista, Australia), accompanied by a stage 4 or 5 behavioural seizure, which was assessed by synchronous video recordings (Curia et al., 2008; Xu et al., 2020). The mice with SRS divided into three groups: NS, sodium valproate (VPA, 200 mg/kg, i.p.) and crocin (100 mg/kg, i.p.) group. Drugs were administrated once daily for 35 days from the observation of the first SRS and an equal volume of NS intraperitoneally was injected into the mice of NS group. The mice in the untreated group were also subjected to the above-mentioned video recording and NS injection. The number of SRS, the duration of each seizure and the seizure stage were measured. The seizure stage was also referenced to the Racine score (Racine, 1972). Some mice, which experienced SE but never observed SRS during the observation period, were not significantly different in seizure severity during SE compared with SRS mice, and were divided into the non-SRS group (nSRS) as a control group in subsequent experiments.

### Open Field Test

The OFT method refers to our previous study (Ye et al., 2017). Briefly, the mice were habituated the laboratory environment 1 day before OFT. Next day, mice was gently placed into the center of the OFT chamber (40 cm × 40 cm × 40 cm, Xinruan, Shanghai, China). Each mouse was allowed to habituate the chamber for 5 min, and then allowed to move freely for 5 min with video recording. The total travelling distance, the travelling distance in the central zone and the duration in the central zone were measured. OFT, which was performed on the same mice as the previous study of SRS, was respectively examined before the pilocarpine-induced SE and after completing all drugs administration.

### Novel Object Recognization Test

The NORT method refers to our previous study (Zhong et al., 2020). Briefly, the mice were habituated the laboratory environment 1 day before NORT. Next day, mice was gently placed into the center of the NORT chamber (40 cm × 40 cm × 40 cm, Xinruan, Shanghai, China). Each mouse was allowed to habituate the chamber for 5 min, and then two identical objects F1 and F2 were placed in the two opposite corners of the field. Then, free movement of each mouse was recorded for 5 min (Test 1, T1), followed by returning the mouse to the cage. After 90 min, F2 was replaced with a new object N (different color from F2), and the mouse was placed into the chamber again for 5 min (Test 2, T2). The behavior of each mouse in T1 and T2 were recorded by video and the software of the NORT chamber was used to analyze the duration of exploring the three objects, and the distance from the tip of the mouse’s nose to the object ≤2 mm was identified as the criterion for exploratory behavior. Discrimination ratio (DR) and discrimination index (DI) was calculated with the following formula. DR = [N or F2/(N or F2 + F1)] × 100%. DI = [(N or F2 - F1)/(N or F2 + F1)]. N, F1, and F2 in the above equations are the duration of exploring each object, respectively. NORT, which was performed on the same mice as the previous study of SRS, was respectively examined before the pilocarpine-induced SE and after completing all drugs administration as well.

### Enzyme Linked Immunosorbent Assay

After the last behavioral test, half of the mice in each group were anesthetized with 1% pentobarbital sodium anesthesia (40 mg/kg, i.p.). Then the animals were sacrificed and the brain tissues were quickly removed. The left and right cortex and hippocampus were separated on the ice, weighed and homogenized in NS at a ratio of 1:9 (w/v), centrifuged at 3000 rpm for 20 min. Then, the supernatants were extracted. The commercially available ELISA kits (Elabscience Biotechnology Co., Ltd., Wuhan, China) for BDNF, TNF-α and IL-1β were used to detect the contents of the three molecules according to the instructions.

### Histology and Neuronal Damage Examination

After the last behavioral test, the other half of the mice in each group were anesthetized with 1% pentobarbital sodium anesthesia (40 mg/kg, i.p.). The animals were sacrificed and the brain tissues were removed following with perfusion through the left ventricle with NS for 20 min and 4% paraformaldehyde for 30 min. The brain tissues were merged in 4% paraformaldehyde for 24 h, and dehydrated with 30% sucrose solution for 2–3 d. The sections were sliced with a thickness of 12 μm on a frozen microtome. After dried, the slices were stained with 1% cresyl violet, and graded with 0.5% hydrochloric acid alcohol, 70%, 90% and 100% alcohol. After decolorization, sealing solution (neutral gum: xylene = 1:1) was used to seal the slices and the hippocampal area was photographed with microscope to compare the neuronal damage in the same area of mice in each group.

At the end of the experiments, electrode placements were histologically verified as well. Brain sections were cut (10 μm) and stained with toluidine blue O. Only the mice with electrodes correctly lying within the hippocampus were included in the statistical analysis. In our experiments, 184 out of 217 mice had electrodes correctly located in the target.

### Statistical Analysis

The statistical analysis was performed using SPSS 17.0 software. Two-way analysis of variance (ANOVA) was performed for repeated measures in the analysis of group differences in kindling and fully kindled model. Comparisons of the number of stimulations for each seizure stage were made with the nonparametric Mann–Whitney U test. The χ2 test was used to compare the incidence of GS and the mortality of pilocarpine-induced SE. Two-way ANOVA was also performed in the analysis of OFT and NORT data. One-way ANOVA was used with Tukey’s t-test in the analysis of other data. Data are presented as mean ± standard error of mean (S.E.M.). *p* < 0.05 indicates a statistical significance.

## Results

### Crocin Retards the Kindling Acquisition in ICR Mice

**FIGURE 2:**
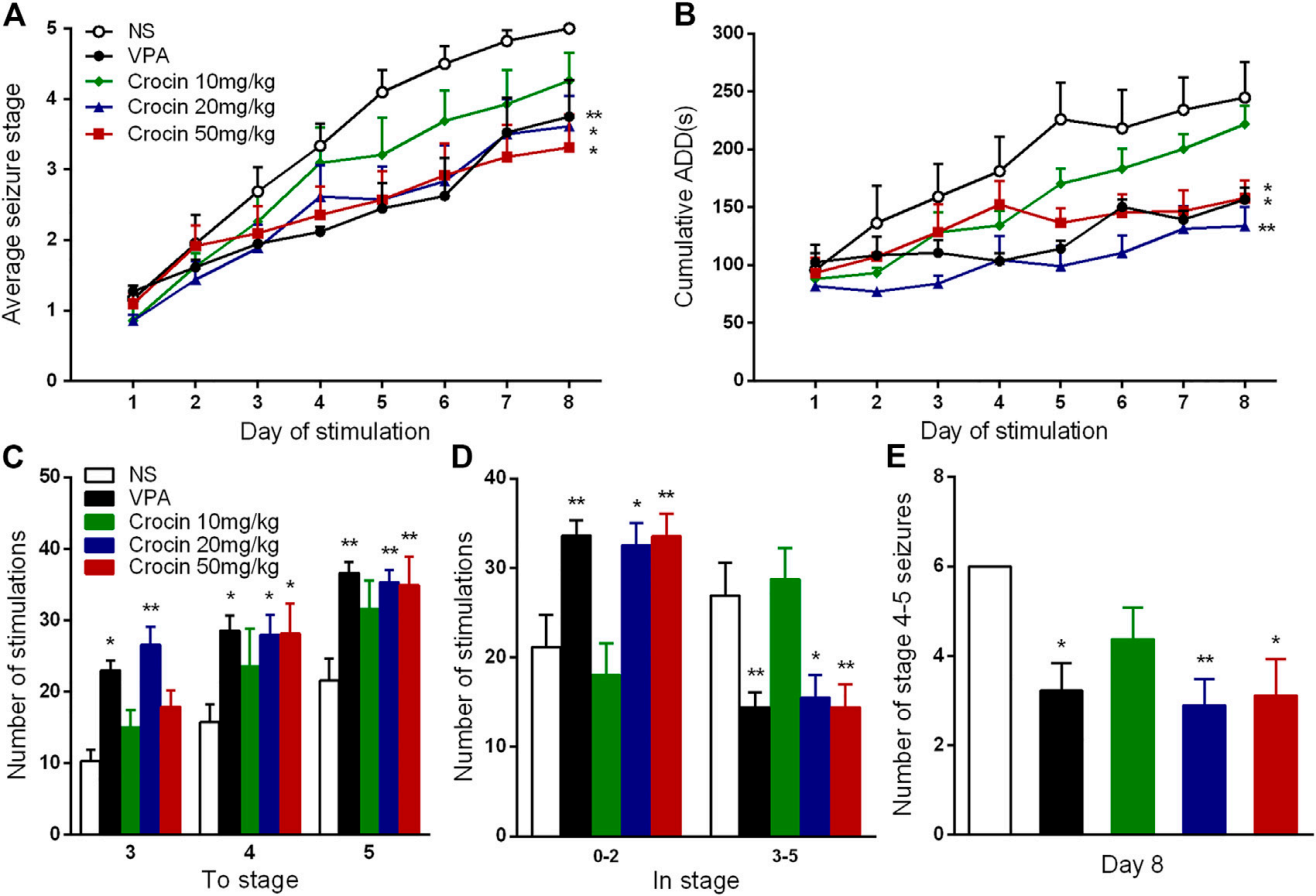
Effects of crocin on hippocampal rapid kindling acquisition (*n* = 8 for each group, x̄±S.E.M.). **(A)** Average seizure stage; **(B)** cumulative ADD of six stimulations each day; **(C)** the number of stimulations to reach seizure stage 3, 4, 5; **(D)** the number of stimulations to stay in seizure stage 0–2 and 3–5 during kindling acquisition; **(E)** Number of stage 4–5 seizures in the 8th day. ADD, afterdischarge duration; NS, normal saline; VPA, sodium valproate. Values are from 3 independent experiments. *p < 0.05, **p < 0.01, compared with NS group. Two-way ANOVA was performed for **(A,B)**. Comparisons of the number of stimulations for each seizure stage **(C,D)** were made with the nonparametric Mann–Whitney U test. One-way ANOVA for repeated measures was used followed by Tukey’s t-test for **(E)**.

Compared with the NS group, 20 and 50 mg/kg crocin significantly retarded the progression of the daily average seizure stages (*p* < 0.05, both, Figure 2A) and shortened the cumulative ADD per day (*p* < 0.01 and *p* < 0.05, Figure 2B). In Figure 2B, 20 mg/kg crocin seemed to be more efffective on reducing the cumulative ADD than 50 mg/kg crocin, although there was not a significant difference. These effects were comparable to that of the positive drug VPA (*p* < 0.01 and *p* < 0.05, Figures 2A,B). Although the effect of 10 mg/kg crocin was yet not significant, it seemed to be a tendency to retard kindling acquisition in the late period of kindling.

To further analyze the possible therapuetic window of crocin, we also calculated the number of stimulations required to each stage and stayed in each stage. Both 20 and 50 mg/kg crocin increased the number of stimulations required to reach the stage 4 (*p* < 0.05, both, Figure 2C) and 5 (*p* < 0.01, both, Figure 2C); meanwhile, the number of stimulations in stage 0–2 was increased (*p* < 0.05 and *p* < 0.01, Figure 2D) and the number of stimulations in stage 3–5 was relatively decreased (*p* < 0.05 and *p* < 0.01, Figure 2D) in 20 and 50 mg/kg crocin group, compared with the NS group. 20 mg/kg crocin also increased the number of stimulations required to reach stage 3 (*p* < 0.01, Figure 2C). In the last 6 kindling stimulations during the last day, the number of stage 4–5 seizures in 20 and 50 mg/kg crocin group was significantly decreased (*p* < 0.01 and *p* < 0.05, Figure 2E), compared with the NS group. These data suggested that crocin may retard the progression of epilepsy in ICR mice.

### Crocin Alleviates the Fully Kindled GS in ICR Mice

**FIGURE 3:**
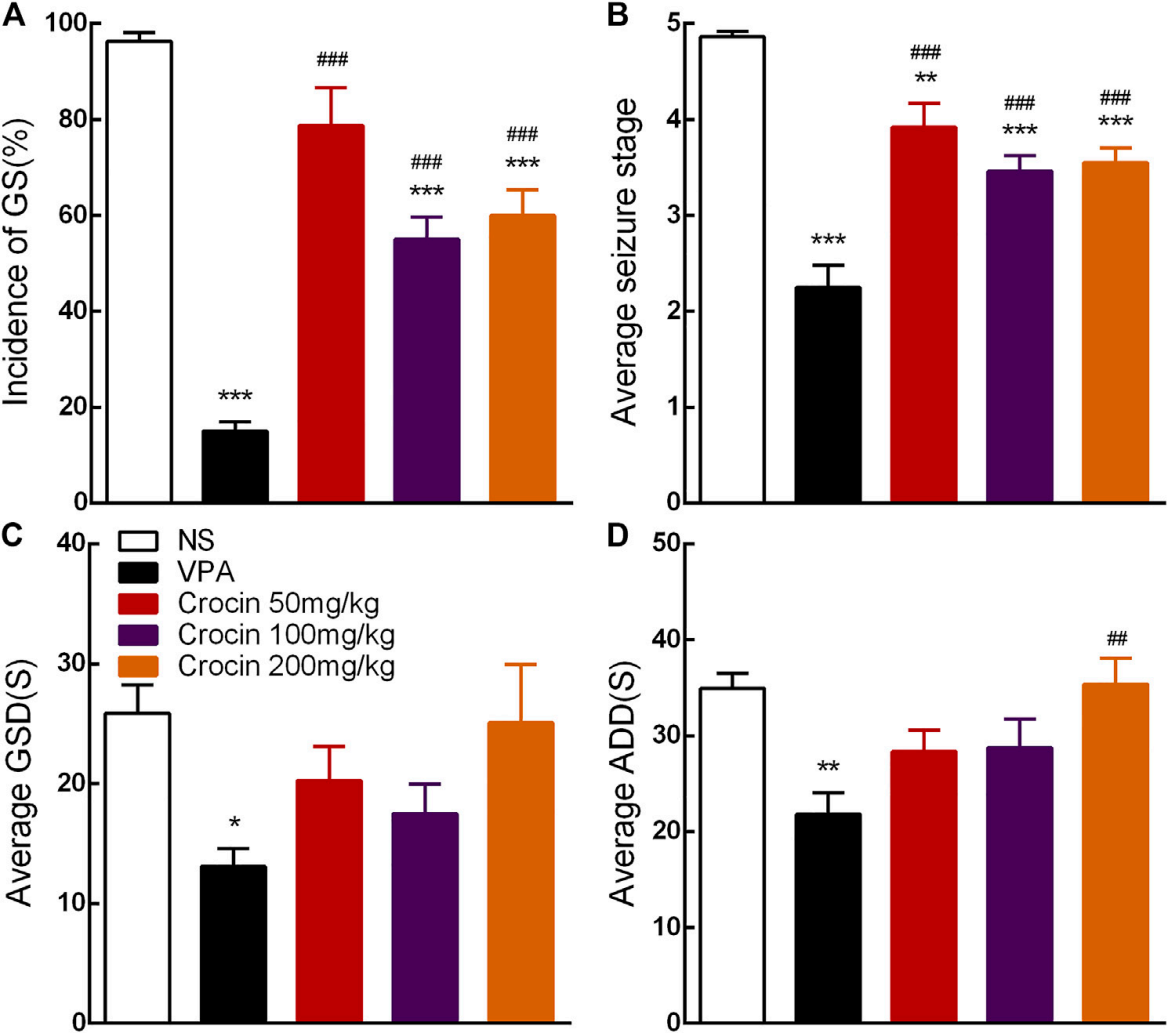
Effects of crocin on the incidence of GS and seizure severity in fully kindled mice (*n* = 8 for each group, x̄±S.E.M.). **(A)** Mean incidence of GS, **(B)** average seizure stage, **(C)** average GSD and **(D)** average ADD during 10 days. GS, generalized seizure, GSD, generalized seizure duration, ADD, afterdischarge duration; NS, normal saline; VPA, sodium valproate. Values are from 3 independent experiments. *p < 0.05, **p < 0.01, ***p < 0.001, compared with NS group; ^##^ *p* < 0.01, ^###^ *p* < 0.001, compared with VPA group. One-way ANOVA was used for **(B–D)**, followed by Tukey’s t-test. The χ2 test was used for **(A)**.

Compared with the NS group, 100 and 200 mg/kg crocin can significantly reduce the incidence of GS (*p* < 0.001, both, Figure 3A) and suppress the average seizure stage (*p* < 0.001, both, Figure 3B), but it did not affect average GSD (*p* > 0.05, Figure 3C) or average ADD (*p* > 0.05, Figure 3D). 50 mg/kg crocin only suppressed the average seizure stage (*p* < 0.01, Figure 3B). However, compared with the VPA group, the inhibitory effect of crocin was relatively weaker (*p* < 0.001, Figures 3A,B and *p* < 0.01, Figure 3D). In addition, neither unexpected behavior nor abnormal EEG was induced by crocin (even the highest dose reached 200 mg/kg) in these mice during the 10 days. These data suggested that crocin at high dose may have an inhibitory effect on the fully kindled GS in ICR mice.

### Crocin Inhibits the Pilocarpine-Induced SE and SRS in ICR Mice

**FIGURE 4:**
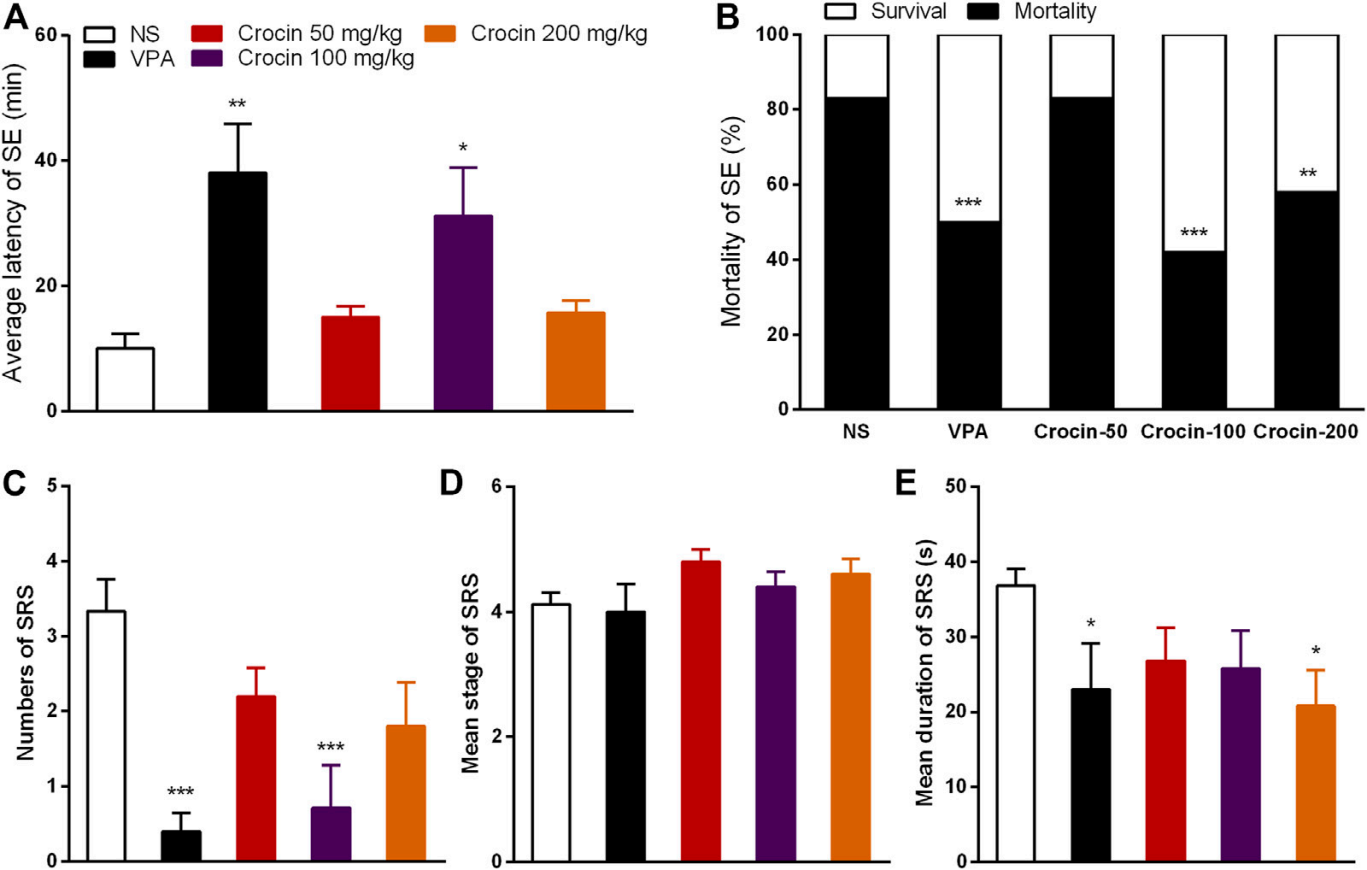
Effects of crocin on pilocarpine-induced SE and SRS. **(A)** average latency of SE (*n* = 12 for each group, x̄±S.E.M.); **(B)** mortality rate of SE (*n* = 12 for each group); **(C)** number of SRS (*n* = 7 for each group, x̄±S.E.M.); **(D)** mean seizure stage of SRS (*n* = 7 for each group, x̄±S.E.M.); **(E)** mean duration of SRS (*n* = 7 for each group, x̄±S.E.M.). SE, status epilepticus; SRS, spontaneous recurrent seizures; NS, normal saline; VPA, sodium valproate. Values are from four independent experiments. *p < 0.05, **p < 0.01, ***p < 0.001, compared with NS group. One-way ANOVA for repeated measures was used followed by Tukey’s t-test for **(A,C,D)** and the χ2 test was used for **(B)**.

To confirm the effect of crocin on SE and SRS, we used the pilocarpine-induced SE and SRS model. In SE model, 100 mg/kg crocin significantly prolonged the latency of SE induced by pilocarpine (*p* < 0.05, Figure 4A) and reduced the mortality of SE (*p* < 0.001, Figure 4B), compared with the NS group. These effects were comparable to that of the positive drug VPA. 200 mg/kg crocin reduced the mortality of SE (*p* < 0.01, Figure 4B), however, 50 mg/kg crocin did not appear any significant effect. In SRS model, compared with the NS group, 100 mg/kg crocin significantly reduced the number of SRS (*p* < 0.001, Figure 4C), which was similar with positive drug VPA (*p* < 0.001, Figure 4C). VPA and 200 mg/kg crocin can reduce mean duration of SRS (*p* < 0.05, Figure 4E). However, neither crocin nor VPA could reduce the mean seizure stage (*p* > 0.05, Figure 4D). These data suggested that crocin at high dose may alleviate SE and SRS.

### Crocin May Not Improve the Cognitive Behaviors After SRS in ICR Mice

**FIGURE 5:**
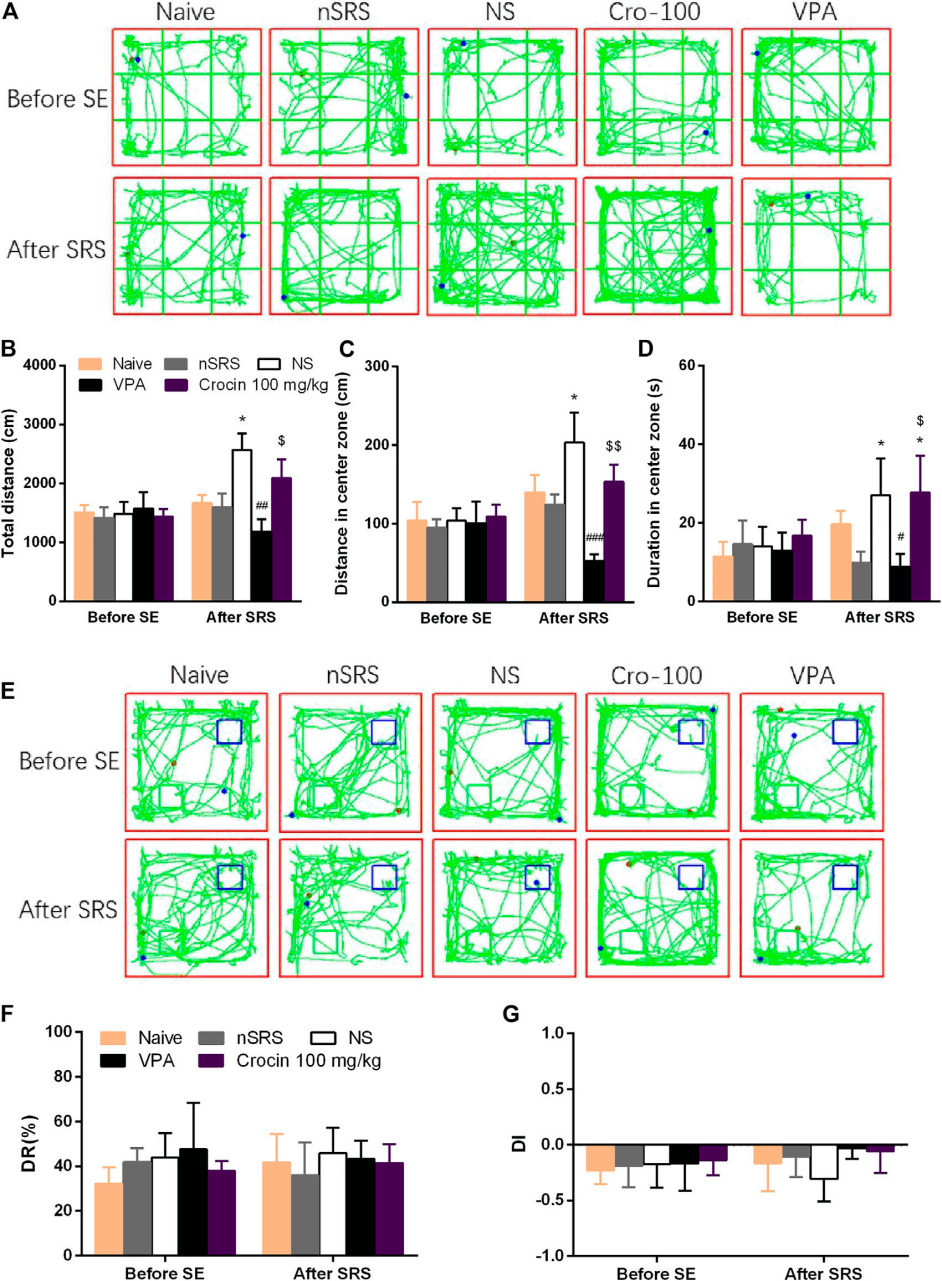
Effects of crocin on the behaviors of mice in OFT and NORT (*n* = 7 for each group, x̄±S.E.M.). **(A)** representative tracks of mice in OFT; **(B)** total travelling distance of mice in OFT; **(C)** movement distance of mice in center zone in OFT; **(D)** duration of mice staying in center zone in OFT; **(E)** representative tracks of mice in NORT (blue boxes mark the location of old object and green boxes mark the location of novel object); **(F)** DR of mice in each group; **(G)** DI of mice in each group. OFT, the open field test; NORT, the novel object recognization test; DR, discrimination ratio; DI, discrimination index; SE, status epilepticus; SRS, spontaneous recurrent seizures; nSRS, non-SRS; NS, normal saline; VPA, sodium valproate. Values are from 3 independent experiments. *p < 0.05, compared with nSRS group; ^#^ *p* < 0.05, ^##^ *p* < 0.01, ^###^ *p* < 0.001, compared with NS group (with SRS); ^$^ *p* < 0.05, ^$$^ *p* < 0.01, compared with VPA group. Two-way ANOVA was performed for **(B,C,D,F,G)**.

To investigate the effect of crocin on the improvement of cognitive function after SRS in mice, NORT was examined. OFT was also performed to assess the movement ability of mice in each group. In the OFT, representative tracks of each group were shown in Figure 5A. The total travelling distance of SRS mice in NS group significantly increased (*p* < 0.05, Figure 5B), compared with that in nSRS group. VPA can significantly decrease the total travelling distance (*p* < 0.01, Figure 5B), compared with that in NS group and crocin (100 mg/kg) had a tendency towards decrease but still higher than that in the VPA group (*p* < 0.05, Figure 5B). The movement distance of mice in the central zone after SRS in NS group was significantly increased than that in nSRS group (*p* < 0.05, Figure 5C). It was significantly reversed in the VPA group (*p* < 0.001, Figure 5C), and 100 mg/kg crocin had a tendency of decline (*p* > 0.05, Figure 5C), compared to the NS group but significantly higher than that in the VPA group (*p* < 0.01, Figure 5C). Similarily, the duration that mice stayed in the central zone after SRS in NS group was significantly increased than that in nSRS group (*p* < 0.05, Figure 5D) and it was only reversed in the VPA group (*p* < 0.05, Figure 5D). These data suggested that the movement of mice may increase after SRS due to their high excitability and VPA but not crocin (100 mg/kg) can significantly reverse the increase.

In the NORT, representative tracks of each group were shown in Figure 5E. As shown, both crocin and VPA had a tendency to improve the DI of mice that suffered SRS, but neither one showed a significant difference (*p* > 0.05, Figure 5G) and they did not improve the DR as well (*p* > 0.05, Figure 5F). These data suggested that crocin at 100 mg/kg may not improve the cognitive behaviors after SRS.

### Crocin Decreases the Loss of Neurons in the Hippocampus After SRS in ICR Mice

**FIGURE 6:**
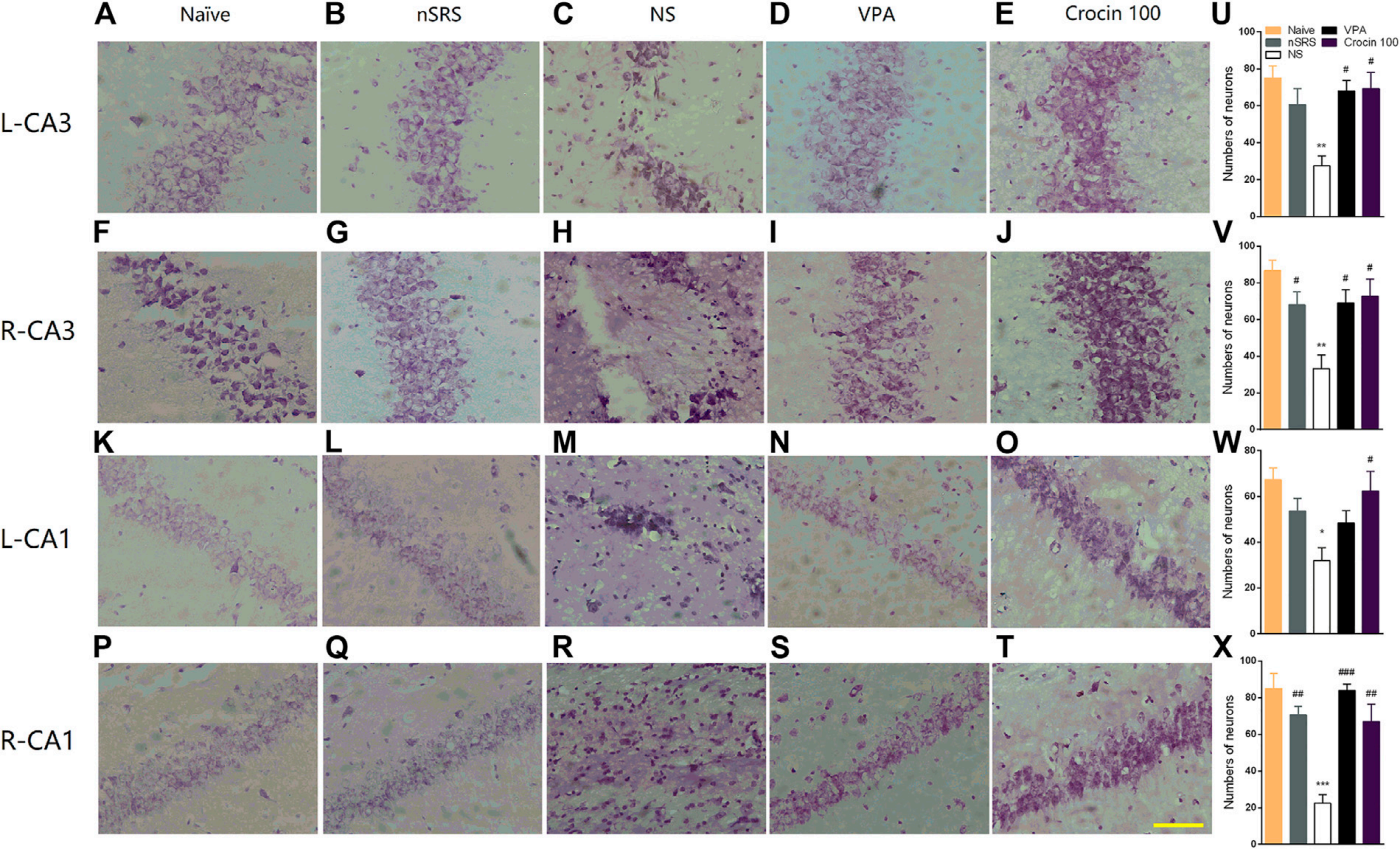
Effects of crocin on the neural damage in the hippocampus region in each group of mice. **(A–E)** H-E staining of neurons in the left hippocampal CA3 region; **(F–J)** H-E staining of neurons in the right hippocampal CA3 region; **(K–O)** H-E staining of neurons in the left hippocampal CA1 region; **(P–T)** H-E staining of neurons in the right hippocampal CA1 region. Bar = 200 μm. **(U–X)** The quantification of survival neurons in left CA3, right CA3, left CA1 and right CA1 (*n* = 3 for each group, x̄±S.E.M.). SRS, spontaneous recurrent seizures; nSRS, non-SRS; NS, normal saline; VPA, sodium valproate. Values are from 3 independent experiments. *p < 0.05, **p < 0.01, ***p < 0.001, compared with naive group; ^#^ *p* < 0.05, ^##^ *p* < 0.01, ^###^ *p* < 0.001, compared with NS group (with SRS). One-way ANOVA was used for **(U–X)**, followed by Tukey’s t-test.

To verify whether crocin has a mitigating effect on neuronal damage after SRS, the neurons in the hippocampus of mice in each group were stained and observed in this experiment. Mice in the NS group showed the loss of neuron in both CA3 and CA1 regions of the right and left hippocampus (Figures 6C,H,M,R), compared to the naïve (Figures 6A,F,K,P) and nSRS group (Figures 6B,G,L,Q); while VPA (Figures 6D,I,N,S) and 100 mg/kg crocin (Figures 6E,J,O,T) treatment ameliorated the neuronal damage. The quantification analysis also showed that the number of survival neurons in the NS group significantly decreased after SRS, compared with that in the naïve group (*p* < 0.01 in left and right CA3, *p* < 0.05 in left CA1, *p* < 0.001 in right CA1, Figures 6U–X) as well as that in nSRS group (*p* < 0.05 in right CA3, Figure 6V; *p* < 0.01 in right CA1, Figure 6X). In VPA and crocin (100 mg/kg) group, more neurons survived in left CA3 (*p* < 0.05 for both VPA and crocin, Figure 6U), right CA3 (*p* < 0.05 for both VPA and crocin, Figure 6V), left CA1 (*p* < 0.05 for crocin, Figure 6W) and right CA1 (*p* < 0.001 for VPA, *p* < 0.01 for crocin, Figure 6X) region of the hippocampus after SRS, compared with that in the NS group. These data suggested that crocin at 100 mg/kg may protect the neurons in CA3 and CA1 region of the hippocampus after SRS.

### Effects of Crocin on the Concentration of BDNF, IL-1β and TNF-α in Brain Tissues of ICR Mice

**FIGURE 7:**
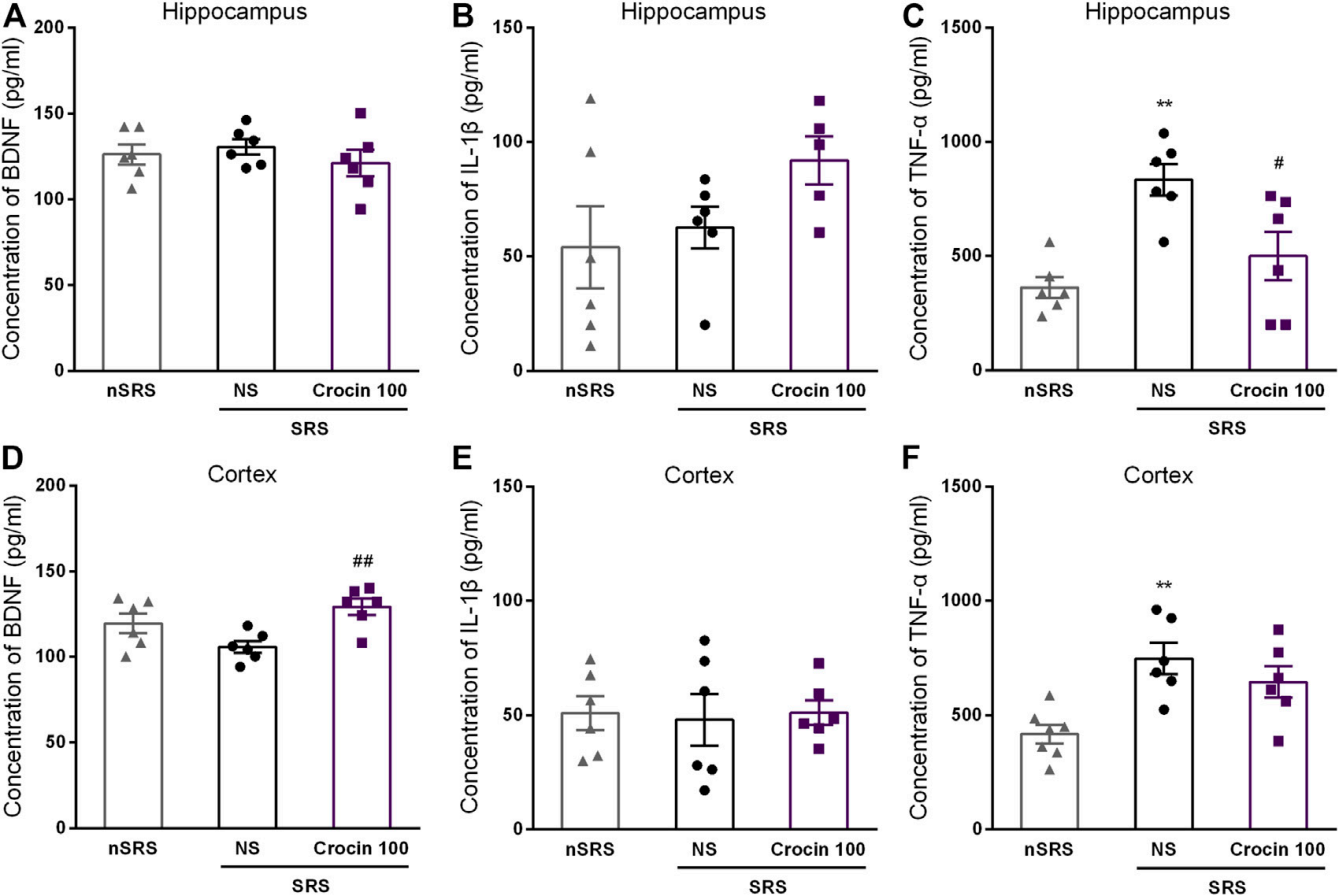
Effects of crocin on intracerebral concentration of BDNF, IL-1β and TNF-α in each group of mice (*n* = 6 for each group, x̄±S.E.M.). **(A)** the concentration of BDNF in the hippocampus of mice; **(B)** the concentration of IL-1β in the hippocampus of mice; **(C)** the concentration of TNF-α in the hippocampus of mice; **(D)** the concentration of BDNF in the cortex of mice; **(E)** the concentration of IL-1β in the cortex of mice; **(F)** the concentration of TNF-α in the cortex of mice. SRS, spontaneous recurrent seizures; nSRS, non-SRS; NS, normal saline; BDNF, brain derived neurotrophic factor; IL-1β, interleukin-1β; TNF-α, tumor necrosis factor-α. Values are from 3 independent experiments. **p < 0.01, compared with nSRS group; #*p* < 0.05, compared with NS group (with SRS). One-way ANOVA for repeated measures was used followed by Tukey’s t-test for **(A–F)**.

To investigate whether the underlying target of crocin is concerned with BDNF, IL-1β or TNF-α, ELISA method was used to measure the concentration of them in the hippocampus and cortex after SRS. Compared with the NS group, 100 mg/kg crocin increased the BDNF concentration in the cortex of SRS mice (*p* < 0.05, Figure 7D), but failed to increase the BDNF concentration in the hippocampus (*p* > 0.05, Figure 7A). In addition, It had little effect on the concentration of IL-1β (*p* > 0.05, Figures 7B,E) in the hippocampus and cortex; however, there was a decrease in the concentration of TNF-α in hippocampus (*p* < 0.05, Figure 7C). These data suggested that crocin at 100 mg/kg may reduce the concentration of TNF-α in hippocampus and increase the concentration of BDNF in the cortex.

## Discussion

In the present study, we mainly found that crocin can alleviate the kindling acquisition and expression in hippocampal rapid electrical kindling model and could suppress the pilocarpine-induced SE and SRS in mice. The protective effect of crocin on neuronal damage may be involved in the inhibition of experimental epilepsy; while the alteration of the concentration of TNF-α in hippocampus and the concentration of BDNF in the cortex may also be one of underlying mechanisms for the anti-epileptic activity of crocin. These results suggested that crocin is effective on the treatment of experimental TLE in mice and it may be a potential anti-epileptic drug.

In our previous study, we have found that crocin has an inhibitory effect on the hippocampal rapid electrical kindling seizures in C57BL/6J mice (Wang et al., 2017a). However, due to the high mortality of the C57BL/6J mice in pilocarpine-induced SRS model, we have to use ICR mice in this study instead. Therefore, we first assessed the effect of crocin on hippocampal rapid electrical kindling seizures in ICR mice to clarify whether or not its effects were strain-specificial. The results illustrated that crocin can alleviate the kindling acquisition and expression in hippocampal rapid electrical kindling model of ICR mice (Figures 2, 3), in accordance with the previous results in C57BL/6J mice (Wang et al., 2017a), which means the effects of crocin are not strain-specificial and it can significantly inhibit the electrical kindling-induced experimental TLE.

In the past researches, the anticonvulsant effects of crocin are controversial. Hosseinzadeh and Talebzadeh (2005) found that crocin did not exhibit anti-epileptic activity against PTZ-induced acute seizure, however, Mazumder et al. (2017) found that crocin can retard the epileptogensis in a PTZ-kindling model of mice. In addition, Tamaddonfard et al. (2012) found that crocin had a significant inhibitory effect on penicillin-induced epileptiform activities. We speculated that there may be two causes for these contradictory results. First, different models of epilepsy were chosen so that the effect of crocin is inconsistent in previous studies, which may suggest the effects of crocin on epilepsy have model selectivity. As the effect of crocin in spontaneous seizure models, which are considered to simulate the characteristics of spontaneous epilepsy in clinic, has not been investigated in previous studies, we mainly use a pilocarpine-induced SRS model to assess the effect of crocin in our present study. The results showed that 100 mg/kg crocin can significantly reduce the number of SRS (Figure 4) and the loss of neuron in the hippocampus (Figure 6), and 200 mg/kg crocin can reduce mean duration of SRS (Figure 4). These data suggested that crocin may be effective against the spontaneous seizures. Second, most previous studies used minor doses or short-term administration (Tamaddonfard et al., 2012; Alavizadeh and Hosseinzadeh, 2014), which may imply that the effect of crocin may be limited by insufficient number of administration or dose of crocin. Therefore, the long-term administration of crocin (daily for 35 days) and higher doses were also chosen in our present study. As we expected, the long-term administration of crocin can inhibit the pilocarpine-induced SRS, however, higher dose (100 mg/kg) rather than the highest dose of crocin showed more effective on pilocarpine-induced SE and SRS. One possible explanation is that crocin has been reported to have multiple acting targets including anti-inflammation, BDNF, and apoptosis etc. (Hosseinzadeh and Talebzadeh, 2005; He et al., 2016; Mazumder et al., 2017; Rana and Musto, 2018; Teng et al., 2021). Due to the multiple targets, different treatment doses may activate different targets and pathways, which may cause the anti-epileptic effect of crocin lacks significant dose-dependence. In addition, the activation of multiple targets may also explain why crocin is effective against multiple types of epileptic model, such as electrical kindling (Figures 2, 3), PTZ kindling (Mazumder et al., 2017), pilocarpine-induced SE and SRS (Figure 4). At present, our data indicated that 100 mg/kg crocin may be the optimal dose and the long-term administration of crocin may be important for inhibiting the SRS. Meanwhile, the animals with crocin did not show any significant behavioral abnormalities during the 35 days, which may be a preliminary evidence of the safety of long-term administration of crocin. In general, our findings provide new and strong evidences to support the view that crocin is effective on epilepsy and it may be a promising anti-epileptic compound, which is worthy of further translational medicine research.

Due to the observed loss of neurons in the hippocampus of SRS mice (Figure 6), we also examined the behaviors of mice in each group by OFT and NORT to observe the influence of the neural damage and possible improvement effect of crocin. In OFT, the activity of SRS mice significantly increased, which may result from the increase in excitability after SRS. VPA can reduce the activity and crocin just has a tendency to reduce the distance of movement in the central zone (Figure 5). In NORT, there were no significant differences in DR and DI among the mice in each group. These data indicated that the crocin may have a weak role of improvement the cognitive behaviors after SRS. We guess that the severity of neuronal damage caused by a few times of SRS was not sufficient to lead to significant cognitive impairment in the mice in this experiment, which is a possible explanation for this result.

The anti-epileptic mechanism of crocin is still unclear. It was thought to be related to enhancement of benzodiazepine receptor system and γ-aminobutyric acid in several previous researches (Mazumder et al., 2017; Rana and Musto, 2018), however, it still exists controversial. For example, the effect of crocin was comparable with diazepam in one report (Rana and Musto, 2018); while diazepam but not equivalent dose crocin was effective on seizures in another report (Hosseinzadeh and Noraei, 2009). So it is probably that there exist other underlying anti-epileptic mechanisms of crocin. Brain-derived neurotrophic factor (BDNF) is a small (14 kD) secreted protein that binds to the ectodomain of its cognate receptor, tyrosin kinase receptor B (TrkB) and low affinity neurotrophic factor receptor. BDNF-TrkB receptor pathway, which plays a major role in triggering a series of downstream cascade reactions, is considered to be closely related with epilepsy (Unsain et al., 2009; Heinrich et al., 2011). As we previously found, crocin can significantly promote the secretion of BDNF in the hippocampus of rats with cerebral ischemia (He et al., 2016). To confirm whether or not crocin can increase the level of BDNF, we measured the concentration of BDNF in hippocampal and cortical regions of mice. Unexpected, it was found that crocin significantly increased the concentration of BDNF in the cortex, but not the hipocampus, of the SRS mice, compared with the NS group (Figure 7). According to previous reports, BDNF plays an important role in the development of epilepsy and is closely related to neuronal survival and apoptosis, brain function and behavior, and synaptic plasticity (Chao, 2003). In recent years, BDNF has also been found to play a role in formation of epilepsy (Walczak et al., 2013). Some studies suggest that BDNF may facilitate or inhibit the process of epilepsy formation (Hong et al., 2013; Chiu et al., 2019), but its exact role and potential mechanisms remain unclear. Pallavi Sharma et al. suggested that the effect of changes in BDNF content on epilepsy formation depends on the brain region where the change occurs and the timing of BDNF cascade pathway activation (Sharma et al., 2019). The results of our experiments support this view. Hence, we hypothesize that increase of BDNF content in the cortex may have an epileptic suppressive effect, and the exact mechanism needs to be further investigated.

In addition, a large number of research reports have already pointed out a correlation between epilepsy and intracranial inflammatory responses (Choi et al., 2009; Riazi et al., 2010; Davis and Dalmau, 2013), where acute or chronic inflammation triggers changes in the content of certain cytokines at specific sites, ultimately leading to neurological dysfunction such as abnormal neuronal excitability, neuronal degeneration and epilepsy (Samland et al., 2003). There are also corresponding immunosuppressive therapies targeting certain inflammatory factors in clinical practice (Vezzani and Baram, 2007). It has been suggested (Dupuis and Auvin, 2015; Dey et al., 2016) that altered levels of inflammatory mediators in the brain will lead to hyperexcitability or damage of neurons and eventually lead to seizures. Then, repeated seizures in turn increase the excitability of neurons to aggravate their damage and death, both of which form a vicious circle and may be a cause of epileptogensis. Similar phenomena have also been found in various SRS animal models. For example, elevated levels of pro-inflammatory cytokines, such as IL-1β, TNF-α, etc., have also been found in the experimental SRS (Shen et al., 2019; Suleymanova, 2021). Based on the above findings, this experiment examined the levels of two pro-inflammatory cytokines, IL-1β and TNF-α, in the hippocampus and cortex. The results showed that the levels of TNF-α in the hippocampus of mice in the crocin group had a decrease, while there was no difference in IL-1β, compared with the NS group (Figure 7). We speculate that crocin may inhibit the inflammatory response through reducing the content of the pro-inflammatory cytokine TNF-α in the hippocampal region, thus achieving the effect of suppressing epilepsy. This is consistent with the findings of Galic et al. and others (Vezzani and Granata, 2005; Galic et al., 2008; D’Ambrosio et al., 2013), who found that anti-TNF-α monoclonal antibodies blocked the proepileptic effects of lipopolysaccharide; moreover, Choi et al. (2009) demonstrated considerable elevation of pro-inflammatory cytokines such as IL-1β in brain tissue of epileptic patients in addition to TNF-α, but these phenomena did not occur in our experiments, which may be related to the regions of the examined brain tissues. In a recent study, elevated levels of IL-1β has also been considered to probably have brain region specificity in a TLE animal model (Xu et al., 2021b). Hence, we will further explore the changes of inflammatory factors in more brain regions in our subsequent experiments. In addition, the relationship between crocin, BDNF and pro-inflammatory cytokines, and whether other signaling molecules are involved in the process also remains to be further investigated.

In conclusion, crocin can significantly retard the progression of epilepsy, alleviate GS and suppress SE and SRS in experimental TLE. Its mechanism may be involved in the protection of neurons, the decrease of TNF-α content in the hippocampus and the increase of BDNF content in the cortex. Thus, crocin may be a potential anti-epileptic compound for treatment of TLE.

## Data Availability

The raw data supporting the conclusion of this article will be made available by the authors, without undue reservation.
